# Bacterial Community Associated with Healthy and Diseased Pacific White Shrimp (*Litopenaeus vannamei*) Larvae and Rearing Water across Different Growth Stages

**DOI:** 10.3389/fmicb.2017.01362

**Published:** 2017-07-18

**Authors:** Yanfen Zheng, Min Yu, Jiwen Liu, Yanlu Qiao, Long Wang, Zhitao Li, Xiao-Hua Zhang, Mingchao Yu

**Affiliations:** ^1^Marine Microbiology Lab, College of Marine Life Sciences, Ocean University of China Qingdao, China; ^2^Tongwei Co., Ltd. Chengdu, China; ^3^Laboratory for Marine Ecology and Environmental Science, Qingdao National Laboratory for Marine Science and Technology Qingdao, China

**Keywords:** bacterial community, *Litopenaeus vannamei* larvae, 454 pyrosequencing, health, disease, growth stages

## Abstract

Bacterial communities are called another “organ” for aquatic animals and their important influence on the health of host has drawn increasing attention. Thus, it is important to study the relationships between aquatic animals and bacterial communities. Here, bacterial communities associated with *Litopenaeus vannamei* larvae at different healthy statuses (diseased and healthy) and growth stages (i.e., zoea, mysis, and early postlarvae periods) were examined using 454-pyrosequencing of the 16S rRNA gene. Bacterial communities with significant difference were observed between healthy and diseased rearing water, and several bacterial groups, such as genera *Nautella* and *Kordiimonas* could also distinguish healthy and diseased shrimp. *Rhodobacteraceae* was widely distributed in rearing water at all growth stages but there were several stage-specific groups, indicating that bacterial members in rearing water assembled into distinct communities throughout the larval development. However, *Gammaproteobacteria*, mainly family *Enterobacteriaceae*, was the most abundant group (accounting for more than 85%) in shrimp larvae at all growth stages. This study compared bacterial communities associated with healthy and diseased *L*. *vannamei* larvae and rearing water, and identified several health- and growth stage-specific bacterial groups, which might be provided as indicators for monitoring the healthy status of shrimp larvae in hatchery.

## Introduction

The intestine of shrimp and their ambient water are both complex ecosystems that harbor diverse bacterial communities, in which some microorganisms are probiotic while some are pathogenic. Microbial dysbiosis might profoundly impact the development and physiological function of their hosts (Whiteson et al., 2014; Rungrassamee et al., 2016; Xiong et al., 2016). Some studies have declared close correlations between the occurrence of shrimp disease and associated bacterial communities (Xiong et al., 2014; Zhang D. et al., 2014). These accumulated knowledge of the complex bacterial communities in aquaculture has refined our perception of which microbial groups could cause diseases. In fact, growing efforts are made to predict the incidence of shrimp disease and find prevention methods from the bacterial perspective (Xiong et al., 2014, 2015; Zhang D. et al., 2014). Xiong et al. (2015) compared the bacterial communities between healthy and diseased shrimps, and found that *Bacilli*, *Flavobacteriales*, *Acidimicrobiales*, and *Alteromonadales* were more abundant in healthy shrimps, whereas *Actinomycetales*, *Sphingobacteriales*, and *Vibrionales* were dominant in diseased shrimps. It was also demonstrated that some bacterial groups (such as *Flavobacteriales* and *Thiotrichales*) could be considered as “health indicators” for predicting shrimp’s health status, and some other bacteria (such as *Rhodobacterales* and *Planctomycetales*) could be considered as “disease indicators" (Zhang D. et al., 2014).

Additionally, some studies have demonstrated that bacterial communities in shrimps varied along with growth stages. Huang et al. (2014) found that *Comamonadaceae* of *Betaproteobacteria* was prevalent in 14-day-old postlarvae (PL14) and 1-month-old juvenile (J1) shrimps, while *Flavobacteriaceae* of *Bacteroidetes* and *Vibrionaceae* of *Gammaproteobacteria* were dominant in 2-month (J2) and 3-month-old juveniles (J3), respectively. Rungrassamee et al. (2013) found *Photobacterium* was the major group in PL15 while *Vibrio* was the dominant group during juvenile stages. Although there were some differences between these two studies, they all found bacterial communities in shrimps shifted along with their development.

However, these previous studies mainly focused on shrimp at juvenile or adult stages, the last two stages in the entire development of shrimp (i.e., egg, larvae, postlarvae, juvenile, and adult). Little is known about shrimps at larval stages including nauplius, zoea, and mysis, when shrimps are susceptible to bacterial diseases due to their underdeveloped digestive and immune systems. For example, the zoea 2 syndrome and mysis mold syndrome were prevalent at zoea and mysis stages, respectively, which would result in mass mortalities in shrimp hatchery (Vandenberghe et al., 1999). Thus, it is very necessary to examine whether there are relationships between the health status of shrimp and the associated bacterial community. The bacterial community associated with larval shrimp has been investigated in a few studies, but they were basically conducted using culture-dependent (Hameed, 1993; Zheng et al., 2016) or fingerprint methods (Pangastuti et al., 2010; Xue et al., 2015). For example, Xue et al. (2015) found that *Flavobacteriaceae* was abundant in rearing water from nauplius 6 to zoea 2 and *Rhodobacteraceae* was the dominant group from zoea 3 to postlarvae using denaturing gradient gel electrophoresis (DGGE) analysis. Our previous study also observed that bacterial communities were changed along with the growth stages of shrimp using culture-dependent methods (Zheng et al., 2016). For excavating the stage-specific bacterial groups in different larval stages in depth, pyrosequencing data is urgently needed.

The purpose of this study was to describe the total bacterial communities in *L. vannamei* larvae (i.e., zoea, mysis, and early postlarvae periods) by 454 pyrosequencing, and attempt to identify the healthy and/or diseased indicators for further application. Total 39 samples were collected from a commercial hatchery, including rearing water samples from ponds with healthy shrimps (WH) and that with diseased shrimps (WD), and shrimp samples from ponds with healthy shrimps (SH) and that with diseased shrimps (SD). The distinct bacterial groups between WH and WD, and between SH and SD were identified by various statistical analyses. Finally, only the bacterial communities in healthy rearing water and shrimp along with different developmental stages were analyzed.

## Materials and Methods

### Rearing of Shrimp Larvae

At zoea stage, live microalgae *Thalassiosira* sp. was used to feed larva for twice daily until they reached zoea 3 stage. After that, brine shrimp (*Artemia*) was added into ponds until postlarvae stage. Shrimp flakes were used at all stages for six times daily. There were no water exchange, antibiotic or commercial probiotics supplement throughout all stages.

### Sample Collection

**FIGURE 1:**
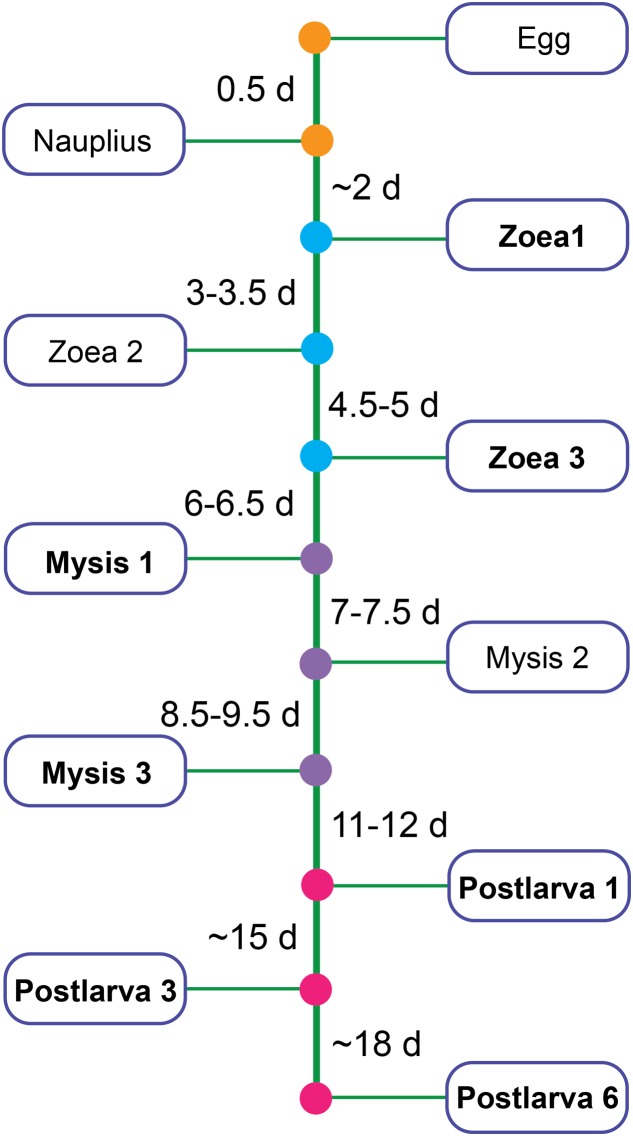
Partial developmental time line of shrimp. Bold type represents the stage chosen to be analyzed in this study.

Shrimps and rearing water were collected from a commercial marine shrimp hatchery from 10 March to 28 April, 2014 in Hainan, China. Healthy shrimp and rearing water were taken from ponds where shrimp larvae had normal feeding behavior, black intestine and/or no apparent sign of disease by visual inspection. Samples covered all key developmental periods: zoea 1 (Z1), zoea 3 (Z3), mysis 1 (M1), mysis 3 (M3), postlarvae 1 (P1), postlarvae 3 (P3), and postlarvae 6 (P6) (a developmental time line was shown in **Figure 1**). Diseased shrimps and rearing water were obtained from ponds where shrimps presented poor growth, inactivity, lack of appetite, empty digestive tracts and/or low survival rate. Shrimp larvae were collected randomly from each pond. Details of the experimental design for sampling was shown in Supplementary Table S1. The surface of shrimp larvae was sprayed with 75% ethanol, and then washed with sterile seawater three times to remove adherent microorganisms. Rearing water was collected with 250 ml sterilized beaker from four different locations in each pond and then pooled. One liter of pooled rearing water was filtered through a 0.22 μm polycarbonate filter (Millipore). All the samples were stored at -80°C for 2 months until DNA extraction.

### DNA Extraction

The whole shrimp larvae (Z1: 200 larvae; Z3: 120 larvae; M1: 80 larvae; M3: 50 larvae; P1: 30 larvae; P3: 20 larvae; P6: 15 larvae) were homogenized using a sterilized glass homogenizer without dissecting intestine due to their small size. The homogenate was mixed with 900 μl of TE buffer (1 M Tris-HCl, 0.5 M EDTA, pH 8.0) and transferred into 2 ml Eppendorf tubes containing 0.3 g quartz sand. The mixture was vigorously beaten on a FastPrep-24 Homogenization System (MP Biomedicals, Irvine, CA, United States) for four times (1 min for each time at a speed of 6.0 m/s), followed by centrifugation at 500 × *g* for 5 min and the supernatant was transferred into a new Eppendorf tube. The following steps and DNA extraction from rearing water were performed according to Yin et al. (2013) with some modification. Briefly, 6 μl of lysozyme (20 mg/ml) were added into each tube, which was incubated at 37°C for 30 min, and then 6 μl of protease K (10 mg/ml) and 60 μl of 10% (w/v) SDS were added and incubated at 65°C for 20 min. Equal amounts of chloroform-isoamyl alcohol (24:1) was used to extract DNA. The supernatant was precipitated with 0.6–0.7 volume isopropanol for 2 h and DNA was resuspended in 50 μl TE buffer.

### PCR Amplification, 454 Pyrosequencing, and Data Analysis

PCR primers 341F and 1073R (sequence was shown in Supplementary Table S2) were selected to amplify the V3–V6 region of the 16S rRNA gene. The PCR reaction system (20 μl) contained 1 × FastPfu Buffer, 2.5 μM of dNTPs, 0.1 μM of each primer, 1 U of FastPfu Polymerase and 10 ng of template DNA. PCR was performed in triplicate at 95°C for 3 min, followed by 27 cycles of 95°C for 30 s, 55°C for 30 s, 72°C for 45 s, and a final extension step of 72°C for 10 min. The triplicate PCR products were combined and purified using an AxyPrep DNA Gel Extraction Kit (Axygen, Hangzhou, China), and then quantified using a Quant-iT PicoGreen double-stranded DNA assay (Invitrogen, Carlsbad, CA, United States). Amplicons from each reaction mixture were pooled with equimolar ratio and subjected to emulsion PCR to generate amplicon libraries. Sequencing was carried out using a Roche Genome Sequencer FLX Titanium platform at Majorbio Bio-Pharm Technology Co., Ltd., Shanghai, China. The produced DNA sequences were processed in QIIME toolkit, version 1.9.1 (Caporaso et al., 2010). Specifically, raw reads were quality filtered and trimmed with Usearch 7.1^1^. Reads completely matching the barcodes and having a maximum single mismatch to the primers were retained. Sequencing adaptor, barcodes and primer sequences were removed. The sequences were further screened by the following thresholds: 0 ambiguous bases, maximum homopolymer stretches of 10 bp, minimum reads length of 200 bp and minimum mean quality score of 20. Several sequences ubiquitous in air, soil and human body and closely related to the potential contaminants, including *Bradyrhizobium*, *Brevundimonas*, *Burkholderia*, *Delftia*, *Erythrobacter*, *Lactococcus*, *Legionella*, *Methylobacterium*, *Mycobacterium*, *Neisseria*, *Novosphingobium*, *Propionibacterium*, *Sphingobium*, *Sphingomonas*, *Sphingopyxis*, *Staphylococcus*, *Stenotrophomonas*, and *Streptococcus* (Nunoura et al., 2015), were removed. Quality-filtered reads were clustered into operational taxonomic units (OTUs) at a 97% similarity level using UPARSE pipeline (Edgar, 2013).

### Quantitative PCR

Quantitative PCR was performed to quantify the abundances of 16S rRNA gene in rearing water and shrimp larvae. The 16S rRNA gene universal primer sets Eub338F/518R (Supplementary Table S2, Yin et al., 2013) were used for quantifying total bacteria. Each 20 μl of quantitative PCR reaction contained the following components: 10 μl of SYBR Green Real-time PCR Master Mix (TaKaRa, Tokyo, Japan), 1 μl of each primer (10 μM), 6 μl of H_2_O, and 2 μl of template DNA. The quantitative PCR was carried out in triplicates. To determine the relationship between PCR cycle threshold (Ct) value and copy numbers, standard curve was obtained by amplifying the 10-fold serially diluted plasmids (pUCm-T, purchasing from Sangon Company, China; the inserted sequence of 16S rRNA gene was shown in Supplementary Table S2), and the copy number of 16S rRNA gene was calculated according to the standard curve. All amplification efficiencies were >99%.

### Statistical Analysis

The alpha diversity index, Chao 1 (Chao and Bunge, 2002) and Shannon estimators (Magurran, 1988) were calculated using Mothur (Schloss et al., 2009). Good’s coverage (Good, 1953) was calculated to evaluate the sampling depth. Linear discriminate analysis (LDA) effect size (LEfSe) (Segata et al., 2011) with default parameters (except for LDA value, which was above 3.0 for rearing water and 2.5 for shrimp larvae) was used to determine bacterial lineages with significant differences (*P* < 0.05) between healthy and diseased samples at various taxonomic levels. Principal component analysis (PCA) was performed by Canoco 5 software at the genus level. Level of statistical significance was determined by *t*-test. Analysis of similarity (ANOSIM) of bacterial communities for different statuses and growth stages of shrimp, and non-metric multidimensional scaling (NMDS) analysis for all samples were carried out using PRIMER 6 (Clarke and Gorley, 2006) based on the Bray-Curtis similarity. The sequence derived from 454 pyrosequencing were deposited in the National Center for Biotechnology Information (NCBI) Short Read Archive database under accession number SRP080243.

## Results

### Samples and Rearing Environment

A total of 39 samples were obtained, including 13 WH, 8 WD, 11 SH, and 7 SD samples (Supplementary Table S1). The parameters of rearing system were shown in **Table 1**.

**Table 1:** The parameters of rearing system.

Parameters	
Initial density	Four million nauplii each pond (∼14000 L)
pH	8.0–8.2
Temperature	30–32°C
Salinity	28–32aaa
Total ammonia nitrogen	<0.2 mg/L
Nitrite nitrogen	<0.1 mg/L

### Overview of 454 Pyrosequencing, Diversity and Abundance of Bacteria

**FIGURE 2:**
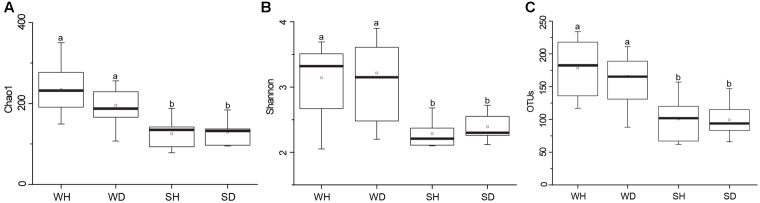
Boxplots of bacterial diversity and richness in WH, WD, SH, and SD. The top and bottom boundaries of each box show the 75th and 25th quartile values, respectively. The black lines within each box represent 50th quartile values. Ends of the whiskers indicate the lowest and highest values. The different letter above the boxes indicate that there is significant difference between groups (*P* < 0.05). **(A,B)** Chao 1 and Shannon indices used to estimate bacterial diversity for each group; **(C)**, OTUs used to determine species richness. WH: water samples from ponds with healthy shrimps, WD: water samples from ponds with diseased shrimps, SH: shrimp samples from ponds with healthy shrimps, SD: shrimp samples from ponds with diseased shrimps.

A total of 563,783 raw reads were obtained by 454 pyrosequencing. The average sequence length was 487 bp. Read numbers of rearing water and shrimp samples ranged from 6272 to 16191 and 16641 to 20634, respectively, of which 6272 and 16641 reads were remained after rarefication. A total of 844 OTUs were obtained, ranging from 88 to 234 and 62 to 157 OTUs in rearing water and shrimp samples, respectively. The Good’s coverages of all samples were greater than 99%, suggesting that the sequencing depth of all samples was sufficient to represent bacterial community in these environments (Supplementary Table S3). Shannon diversity index showed variations of 2.05–3.67 in WH, 2.20–3.90 in WD, 2.10–2.68 in SH, and 2.12–2.72 in SD, respectively. The Chao 1 index of WH, WD, SH and SD samples were 149–350, 107–256, 78–188, and 95–184, respectively (Supplementary Table S3). Chao 1 and Shannon indices of water samples were higher than those of shrimp samples (*P* < 0.05), whereas showed no significant differences between WH and WD (*P* > 0.05), and between SH and SD (*P* > 0.05) (**Figure 2**). Similarly, there was also no significant difference in the number of OTUs between WH and WD, and between SH and SD (**Figure 2**).

According to the result of quantitative PCR, the bacterial 16S rRNA gene abundance ranged from 1.5 × 10^6^ to 4.7 × 10^7^ copies/ml in water and 2.4 × 10^7^ to 3.1 × 10^9^ copies/g in shrimp larvae. There was significant difference (*P* < 0.05) of 16S rRNA gene abundance in the rearing water between zoea (1.5 × 10^6^–6.9 × 10^6^ copies/ml) and mysis (7.3 × 10^6^–3.0 × 10^7^ copies/ml) stages, but no significant difference (*P* > 0.05) was observed between mysis and postlarva (6.0 × 10^6^–4.7 × 10^7^ copies/ml) stages (Supplementary Figure S1). The 16S rRNA gene copy numbers in shrimp increased with growth stages, but with no significant difference (Supplementary Figure S1).

### Distinct Bacterial Groups between WH and WD, and between SH and SD

**FIGURE 3:**
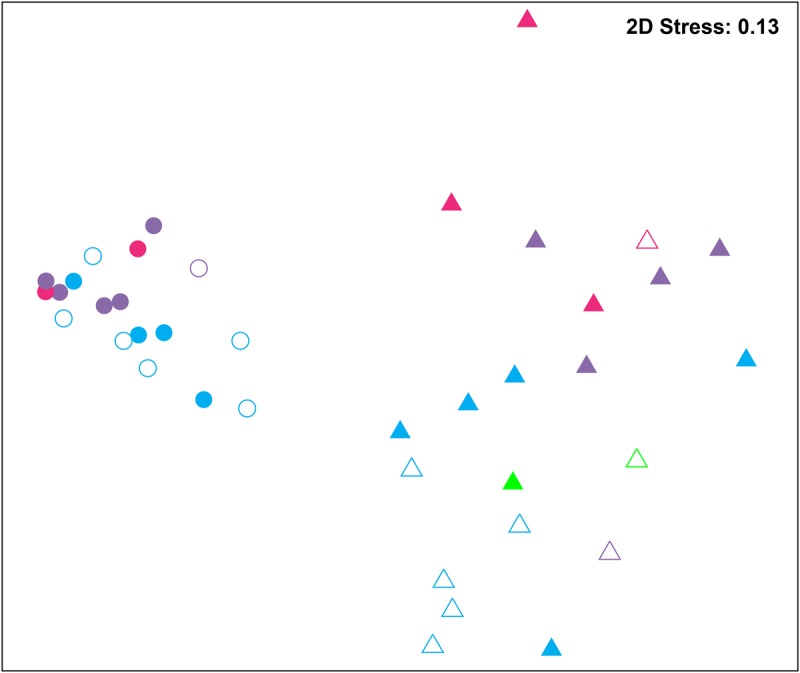
Non-metric multidimensional scaling (NMDS) analysis of all samples based on OTU level. Bray–Curtis similarity metric was used with PRIMER 6. Circle and triangle represent shrimp and water samples, respectively. Blue, purple, and red represent zoea, mysis, and postlarvae stages, respectively. Green: rearing water before larvae were released into pond. Symbols filled and unfilled color indicated healthy and diseased, respectively.

**FIGURE 4:**
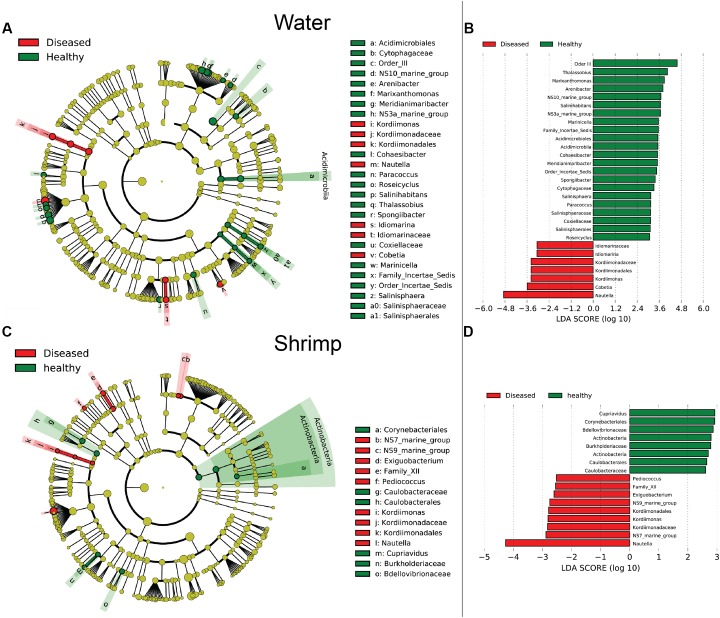
The cladograms of bacterial lineages with significant difference between WH and WD, and between SH and SD. The bacterial groups from phylum to genus level are listed from center to outside. Each circle’s diameter is proportional to the bacterial taxon’s abundance. Green: bacterial taxa enriched in healthy water or shrimp; red: bacterial taxa enriched in diseased water or shrimp; yellow: no significant differences. **(A,C)** Only the taxa that linear discriminate analysis (LDA) value above 3.0 for rearing water and 2.5 for shrimp larvae are shown, respectively. **(B,D)** The length of column represented the effect size of bacterial lineages.

Bacterial communities were compared among all samples using NMDS analysis at the OTU level. Bacterial communities in rearing water were separated from shrimp (**Figure 3**). The low similarity (**Figure 3**) of bacterial communities between WH and WD was confirmed by the result of ANOSIM (*r* = 0.281, *P* = 0.003), suggesting that bacterial communities were distinct between healthy and diseased water. Based on the above analyses, we used LEfSe to find the potential discriminating taxa between healthy and diseased water. The results showed that there were 31 bacterial taxa distinguishing WD from WH with LDA value greater than 3.0 (**Figure 4A**). One class, 4 orders, 4 families, and 13 genera were enriched in WH, including *Acidimicrobiia* (from class to order levels), *Salinisphaerales* (from order to genus levels), Order Incertae Sedis (from order to genus levels), Order III, *Cytophagaceae* (family level) and *Coxiellaceae* (family level). Moreover, there were many groups only enriched at genus level, including *Arenibacter*, *Cohaesibacter*, *Marixanthomonas*, *Meridianimaribacter*, NS10 marine group, NS3a marine group, *Paracoccus*, *Roseicyclus*, *Salinihabitans*, *Spongiibacter*, and *Thalassobius* (**Figures 4A,B**). In WD samples, one order, two families and four genera were enriched, including *Kordiimonadales* (from order to genus levels), *Idiomarinaceae* (from family to genus levels), *Cobetia* and *Nautella*.

Although SH and SD could not be separated from NMDS analysis (**Figure 3**) and no significant difference (*r* = 0.169, *P* = 0.055) was observed as well, there were still several bacterial taxa which could distinguish these two groups by LEfSe. One phylum, one class, two orders, three families, and one genus were enriched in SH, including *Actinobacteria* (from phylum to class levels), *Caulobacterales* (from order to family levels), *Corynebacteriales* (order level), *Bdellovibrionaceae* (family level), and *Burkholderiaceae* (from family to genus levels), while one order, four families, and four genera were enriched in SD, including *Kordiimonadales* (from order to genus levels), Family XII, NS7 marine group (family level), NS9 marine group (family level) and genera *Exiguobacterium*, *Pediococcus*, and *Nautella* (**Figures 4C,D**). Interestingly, the genus *Nautella* in diseased rearing water and shrimps both showed the largest effect size (LDA value > 4.0) (**Figures 4B,D**). The relative abundance of *Nautella* in WH, WD, SH, and SD were 6.19, 24.68, 0.19, and 3.00%, respectively (Supplementary Figure S2).

**FIGURE 5:**
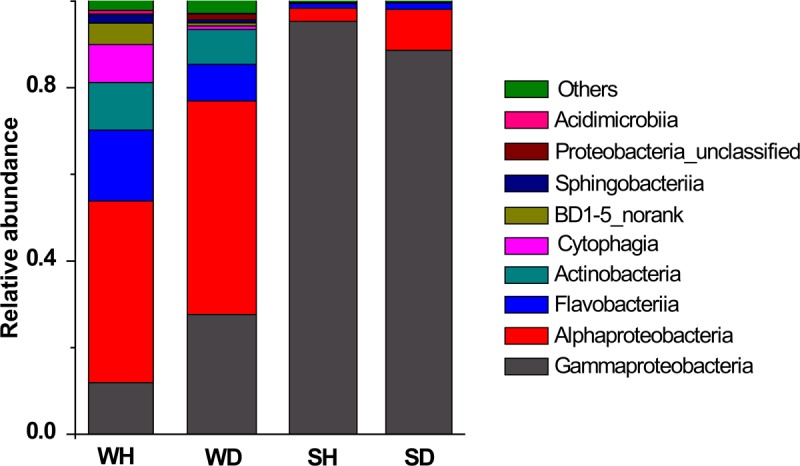
Bacterial composition associated with WH, WD, SH, and SD (based on class level). WH: water samples from ponds with healthy shrimps, WD: water samples from ponds with diseased shrimps, SH: shrimp samples from ponds with healthy shrimps, SD: shrimp samples from ponds with diseased shrimps.

Four classes (*Alphaproteobacteria*, *Gammaproteobacteria*, *Flavobacteriia*, and *Actinobacteria*) were shared in WH and WD, but varied in their relative abundance (**Figure 5**). There were more *Flavobacteriia* and *Cytophagia* in WH while the abundance of *Gammaproteobacteria* and *Alphaproteobacteria* increased in WD. *Gammaproteobacteria* was always the overwhelming bacterial groups in shrimp, but the relative abundance of *Alphaproteobacteria* is lower in SH than that in SD (**Figure 5**).

### Different Bacterial Communities along with Growth Stages

**FIGURE 6:**
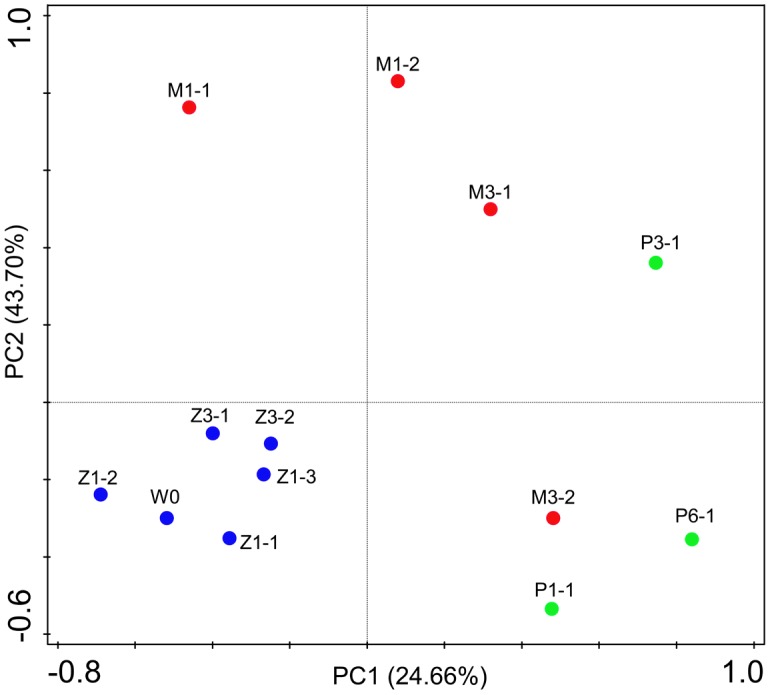
Principal component analysis (PCA) plot of rearing water under different growth stages at family level. W0: water before larvae were released into pond. Z, zoea; M, mysis; P, postlarvae.

**FIGURE 7:**
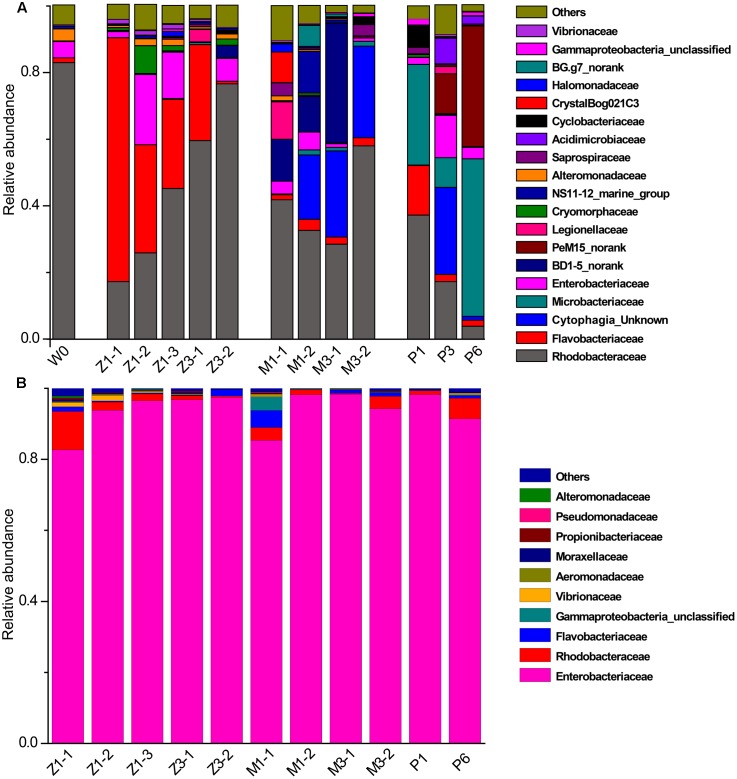
Relative abundance of the dominant bacterial families in rearing water and shrimp. **(A)** Rearing water; **(B)** shrimp. W0: water before larvae were released into pond; Z, zoea; M, mysis; P, postlarvae.

Bacterial communities along with growth stages were analyzed to study the changing tsrend they followed throughout the key developmental stages and to find out the stage-specific groups. PCA at family level (**Figure 6**) showed that bacterial groups in the rearing water under different growth stages were clustered separately, which was also confirmed by ANOSIM analysis (*P* < 0.05) (**Table 2**). The PC1 axis (24.66%) discriminated the zoea from postlarvae stage, while the PC2 axis (43.70%) discriminated the mysis from zoea and postlarvae stages except for sample M3-2. *Rhodobacteraceae* was abundant in rearing water at all tested growth stages, whereas its relative abundance displayed a decreasing trend at mysis and postlarva stages (**Figure 7**). Meanwhile, some bacterial groups exhibited stage-specific signatures. Specifically, *Flavobacteriaceae* was abundant at zoea stage compared with that at mysis stage (*P* < 0.05). Subsequently, its abundance decreased and BD1-5 clade of *Actinobacteria* increased at mysis stage (*P* < 0.05). At postlarva stage, *Microbacteriaceae* (phylum *Actinobacteria*) increased being the dominant bacterial group (*P* < 0.05) (**Figure 7**). Detailed bacterial community composition of water samples at genus level was exhibited by heatmap (Supplementary Figure S3). An unclassified genus of *Rhodobacteraceae* was predominant in all rearing water samples. Although *Nautella* was prevalent at zoea stage, its abundance decreased at mysis and postlarva periods (Supplementary Figure S3).

**Table 2:** Differences of bacterial community of rearing water at different growth stages assessed by ANOSIM tests based on family level.

Growth stages	Zoea	Mysis	Postlarva
Zoea			
Mysis	*r* = 0.600, *P* = 0.016		
Postlarva	*r* = 0.631, *P* = 0.018	*r* = 0.556, *P* = 0.029	

*Bold type (*P* < 0.05) indicates significant difference between two groups. Correlation (*r*) and significance (*P*) values are shown*.

**FIGURE 8:**
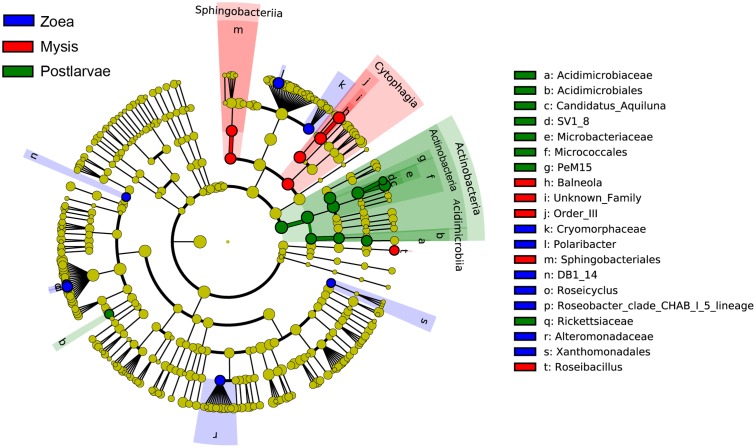
The cladograms of bacterial lineages with significant difference in rearing water under different growth stages. The bacterial groups from phylum to genus level are listed from center to outside. Each circle’s diameter is proportional to the bacterial taxon’s abundance. Blue: bacterial taxa enriched in zoea stage; red: bacterial taxa enriched in mysis stage; Green: bacterial taxa enriched in postlarva stage; yellow: no significant differences. Only the taxa that LDA value above 3.0 are shown.

A total of 25 bacterial groups were found to have significant differences along with different growth stages using LEfSe. Two orders, two families and three genera were enriched at zoea stage, including *Xanthomonadales* (order level), DB1-14 (order level), *Cryomorphaceae* (family level), *Alteromonadaceae* (family level), *Polaribacter* (genus level), *Roseicyclus* (genus level), and *Roseobacter* clade CHAB-l-5 lineage. Two classes, two orders, one family, and two genera were enriched at mysis stage, including *Cytophagia* (from class to genus), *Sphingobacteriia* (from class to order), and *Roseibacillus* (genus level). One phylum, two classes, three orders, three families, and two genera were enriched at postlarvae stage, including *Actinobacteria* (from phylum to genus) and *Rickettsiaceae* (genus level) (**Figure 8**).

By contrast, little variation of bacterial community in shrimp was observed along with the growth of shrimp. *Enterobacteriaceae* of *Gammaproteobacteria* was the most abundant group, accounting for more than 85% at all growth stages (**Figure 7**). Correspondingly, *Enterobacter* and some unclassified genera of *Enterobacteriaceae* was the most abundant genera in shrimp at all growth stages, followed by an unclassified genus of *Rhodobacteraceae*, then genera *Ruegeria*, *Aquimarina*, and *Vibrio*. These results indicated that bacterial community change in the rearing water only have limited influence on that of shrimp larvae.

## Discussion

Bacterial communities in juvenile shrimps have been described extensively (Rungrassamee et al., 2013; Huang et al., 2014; Zhang D. et al., 2014; Xiong et al., 2015), but in larval shrimp it is poorly understood. Here, we compared bacterial communities associated with healthy and diseased *L. vannamei* larvae and the related rearing water along with shrimp development. The results showed that distinct bacterial communities assembled between healthy and diseased water, indicating that some specific bacterial groups might be applied as indicators for monitoring the health status of shrimp larvae in hatchery.

The intestine of aquatic animals and rearing water were reported to be fertile grounds for various microorganisms. Hameed (1993) observed that the total culturable bacterial count of *Penaeus indicus* larval rearing water ranged from 9.0 × 10^2^ to 1.0 × 10^5^ cfu/ml. In this study, the bacterial 16S rRNA gene abundance ranged from 1.5 × 10^6^ to 4.7 × 10^7^ copies/ml in the rearing water. It was reported that the average number of 16S rRNA gene copies in one bacterium is 4.14 (Lee et al., 2009). Thus, there were ∼3.6 × 10^5^ to 1.1 × 10^7^ bacteria/ml rearing water in our study, which was approximately two orders of magnitude higher than the results based on culture-dependent method (Yasuda and Kitao, 1980; Hameed, 1993; Kennedy et al., 2006). It was also confirmed the idea that most of the bacteria in environment were hard to cultivate. Furthermore, in this study, there were ∼5.8 × 10^6^ to 7.6 × 10^8^ bacteria/g larvae, higher than the number of Hameed’s (1993) results that cultivable bacterial counts ranged from 8.1 × 10^4^ to 1.2 × 10^8^ cfu/g at larval stage.

Comparing the healthy and diseased rearing water samples, we found that more *Flavobacteriia* and *Cytophagia* in WH while more *Gammaproteobacteria* and *Alphaproteobacteria* in WD (**Figure 5**). Xiong et al. (2015) also demonstrated that *Flavobacteriia* and *Gammaproteobacteria* was abundant in healthy and diseased shrimp, respectively. In fact, *Flavobacteriia* was reported to have a specialized ability in degrading complex organic matter and biopolymers such as cellulose and chitin (Kirchman, 2002; Williams et al., 2013), implying that members of this bacterial taxa might have positive effect on improving rearing water quality. It was reported that high abundance of *Gammaproteobacteria* presented in diseased shrimps was attributed to *Vibrio* (Rungrassamee et al., 2016). Unexpectedly, *Vibrio* was rarely detected in diseased water and shrimp in this study, which was consistent with the results of Zhang D. et al. (2014) that also observed low and almost unchanged relative abundance of *Vibrio* in diseased shrimp. At times there appears to be no close relationship between the emergence of disease and the abundance of *Vibrio* (Sung et al., 2001).

Although bacterial communities at high taxonomic levels have no difference between SH and SD, several distinguished bacterial groups were identified from LEfSe analysis. Specially, the genus *Nautella* with the largest effect size (LDA value higher than 4.0) was enriched in both diseased rearing water and shrimp (**Figure 4**). Sakami et al. (2014) reported that *Nautella* was common in rotifer culture tanks, but there were other studies found that bacteria in this genus were pathogenic toward red alga *Delisea pulchra* (Gardiner et al., 2015) and brine shrimp (*Artemia*) (Zheng et al., 2016). Therefore, *Nautella* might be provided as a diseased indicator for monitoring the health of shrimp. Additional experiments are suggested to prove whether there is a close relation between the health of shrimp and *Nautella*. Several bacterial groups were enriched in healthy water and shrimp, such as genus *Meridianimaribacter* (**Figure 4** and Supplementary Figure S2). Member in genus *Meridianimaribacter* was frequently found in both healthy water and shrimp in our previous study using culture-dependent method (Zheng et al., 2016). *Meridianimaribacter* was also found in the intestinal tract of shrimp after adding probiotics (Luis-Villaseñor et al., 2013). It was reported that *Meridianimaribacter* was dominant in healthy larviculture water of shrimp (Xue et al., 2015). Possibly, bacteria in *Meridianimaribacter* have a beneficial effect on the health of its hosts and we propose that it might be considered as a probiotic candidate and an indicator of healthy status in shrimp larvae aquaculture.

The bacterial community of larval rearing water was primarily dominated by *Rhodobacteraceae* of *Alphaproteobacteria*, agreed with previous studies (Huang et al., 2014; Xue et al., 2015). *Rhodobacteraceae* may act as the keystone species in rearing water and may have a potential interaction with shrimp at different growth stages, which need to be further characterized. Several bacterial groups exhibited different relative abundance in different growth stages. *Flavobacteriaceae* (phylum *Flavobacteriia*), BD1-5 clade (class) and PeM15 clade (order) (phylum *Actinobacteria*), and *Microbacteriaceae* (phylum *Actinobacteria*) have a relative high abundance at the zoea, mysis and postlarvae periods, respectively, which might be stage-specific bacterial groups. Several studies demonstrated that diets had influence on the bacterial communities of shrimp (Huang et al., 2014; Zhang M. et al., 2014). In this study, shrimp at zoea stage were fed with microalgae *Thalassiosira* sp. until they reached zoea 3 stage. After that, the diet was changed to *Artemia*. This shift of diet from microalgae to *Artemia* might explain the variance of bacterial composition in rearing water with growth stages.

Contrastingly, *Gammaproteobacteria* was the most abundant group in shrimp, which was in accordance with that in other shrimp species (Liu et al., 2011; Chaiyapechara et al., 2012; Rungrassamee et al., 2013) and other aquatic animals including fish (Verner-Jeffreys et al., 2003; McIntosh et al., 2008), shellfishes (Payne et al., 2007; Meziti et al., 2010) and bivalves (Sandaa et al., 2003; Tanaka et al., 2004). Consistent with the previous study of Chaiyapechara et al. (2012), our results revealed that bacterial community in shrimp was different from that in rearing water as well. At a finer taxonomic level, we found that *Enterobacteriaceae* of *Gammaproteobacteria* was dominant in shrimp while *Vibrionaceae* or other families were abundant in other studies (Rungrassamee et al., 2013, 2014; Huang et al., 2014). It has been documented that members of *Enterobacteriaceae* were abundant in digestive tract of freshwater and marine fish (Ringø and Birkbeck, 1999; Merrifield et al., 2009; Wang et al., 2014) and healthy pigs (Schierack et al., 2007), but not in shrimps. In general, bacteria in this family frequently attached to fecal matter in intestine. In other studies, shrimp intestines were dissected and the residue inside was removed before genomic DNA extraction; however, the whole shrimp larvae were used in our study due to their small size. We speculated that the fecal matter in larval intestine contributed to the high abundance of *Enterobacteriaceae*.

In general, our results revealed that bacterial members of rearing water assembled into distinct communities along with growth stages but showed little variation in shrimp. Different diets at different stages might explain the variance of bacterial composition in rearing water. Further studies are needed to confirm the observation with *L*. *vannamei* larvae of this study in other hatcheries or other shrimp species. Overall, the present study built significant relationships among shrimp larva at two healthy statuses (healthy and diseased) and growth stages, which may provide instructional insights for using specific bacterial groups to indicate healthy status. In the future, we are supposed to take new strategies toward predicting diseases rather than only focusing on how to treat them. Certainly, we cannot eliminate the possibility that diseases caused from other types of organisms, such as fungi and viruses.

## Ethics Statement

This study was carried out in accordance with the recommendations of Animal Ethics Committee of Shandong Province, China. The protocol was approved by the Animal Ethics Committee of Shandong Province, China.

## Author Contributions

X-HZ and McY designed the study. YZ did the experiment and wrote the manuscript with assistance of MnY and JL. YQ and LW helped to analyze the data and revise the manuscript. ZL contributed to collect samples from shrimp hatchery. All authors approved the final manuscript.

## Conflict of Interest Statement

The authors declare that the research was conducted in the absence of any commercial or financial relationships that could be construed as a potential conflict of interest.

## Footnotes

1 http://drive5.com/uparse/
