# Supplementary material for: Bacterial Community Associated with Healthy and Diseased Pacific White Shrimp (Litopenaeus vannamei) Larvae and Rearing Water across Different Growth Stages
Source: Front Microbiol. 2017 Jul 18;8:1362. doi: 10.3389/fmicb.2017.01362 (PMC5513922; doi:10.3389/fmicb.2017.01362)
Supplement: Supplementary file 1 [file Data_Sheet_1.DOCX]

**Supplementary materials for**

**Bacterial Community Associated with** **Healthy and Diseased Pacific White Shrimp (*Litopenaeus vannamei*) Larvae and Rearing Water across Growth Stages**

Yanfen Zheng^1,†^, Min Yu^1,†^, Jiwen Liu^1^, Yanlu Qiao^1^, Long Wang^1^, Zhitao Li^2^, Xiao-Hua Zhang^1,3*^, Mingchao Yu^2*^

^1^ College of Marine Life Sciences, Ocean University of China, Qingdao 266003, China

^2^ Tongwei Co., LTD, Chengdu, 610093, China

^3^ Laboratory for Marine Ecology and Environmental Science, Qingdao National Laboratory for Marine Science and Technology, Qingdao 266071, China

† These authors contributed equally to this work.

Table S1 The details of the experimental design for sampling. Samples were not pooled together from different tanks. Each sample was from one tank. W0: water before larvae were released into pond; Z: zoea; M: mysis; P: postlarvae.

| Healthy status | Water samples | Water collected | Water used to extract DNA | Shrimp samples | Larvae collected | Larval number used to extract DNA |
| --- | --- | --- | --- | --- | --- | --- |
| Healthy | W0 | 2 L | 500 ml | - | - | - |
|  | Z1-1 | 2 L | 500 ml | Z1-1 | ~1000 | ~200 |
|  | Z1-2 | 2 L | 500 ml | Z1-2 | ~1000 | ~200 |
|  | Z1-3 | 2 L | 500 ml | Z1-3 | ~1000 | ~200 |
|  | Z3-1 | 2 L | 500 ml | Z3-1 | ~1000 | ~120 |
|  | Z3-2 | 2 L | 500 ml | Z3-2 | ~1000 | ~120 |
|  | M1-1 | 2 L | 500 ml | M1-1 | ~500 | ~80 |
|  | M1-2 | 2 L | 500 ml | M1-2 | ~500 | ~80 |
|  | M3-1 | 2 L | 500 ml | M3-1 | ~500 | ~50 |
|  | M3-2 | 2 L | 500 ml | M3-2 | ~500 | ~50 |
|  | P1-1 | 2 L | 500 ml | P1-1 | ~100 | ~30 |
|  | P3-1 | 2 L | 500 ml | - | - | - |
|  | P6-1 | 2 L | 500 ml | P6-1 | ~100 | ~15 |
| Diseased | W0 | 2 L | 500 ml | - | - | - |
|  | Z1-1 | 2 L | 500 ml | Z1-1 | ~1000 | ~200 |
|  | Z1-2 | 2 L | 500 ml | Z1-2 | ~1000 | ~200 |
|  | Z2-1 | 2 L | 500 ml | Z2-1 | ~1000 | ~150 |
|  | Z2-2 | 2 L | 500 ml | Z2-2 | ~1000 | ~150 |
|  | Z3-1 | 2 L | 500 ml | Z3-1 | ~1000 | ~120 |
|  | M3-1 | 2 L | 500 ml | M3-1 | ~500 | ~50 |
|  | P1-1 | 2 L | 500 ml | P1-1 | ~100 | ~30 |

Table S2 Primers and inserted sequence used in the study.

| **Name** | **Sequence (5’-3’ direction)** | **Usage** |
| --- | --- | --- |
| 341F | CCTACGGGAGGCAGCAG | Primer in 454 Pyrosequencing |
| 1073R | ACGAGCTGACGACARCCATG |  |
| Eub338F | ACTCCTACGGGAGGCAGCAG | Primer in qPCR |
| Eub518R | ATTACCGCGGCTGCTGG |  |
| The inserted sequence of 16S rRNA gene of plasmid | ACTCCTACGGGAGGCAGCAGTGAGGAATATTGGACAATGGAGGCAACTCTGATCCAGCCATGCCGCGTGCAGGATGACGGCCCTATGGGTTGTAAACTGCTTTTATGTAGGAAGAAACACTCTCACGTGTGAGAGCTTGACGGTACTACAAGAATAAGGACCGGCTAACTCCGTGCCAGCAGCCGCGGTAATA | Standard in qPCR |

Table S3 Summary of quality filtered reads, bacterial richness (OTUs), bacterial diversity indices (Chao 1 and Shannon) and sample coverage (Good’s coverage) of all samples in the study.

| **Water**  Samples | Reads | OTUs | Chao I | Shannon | Good’s | **Shrimp**  Reads | OTUs | Chao I | Shannon | Good’s |
| --- | --- | --- | --- | --- | --- | --- | --- | --- | --- | --- |
| **Healthy** |  |  |  |  |  |  |  |  |  |  |
| W0 | 6272 | 164 | 202 | 3.07 | 0.99 | - | - | - | - | - |
| Z1-1 | 6272 | 181 | 234 | 3.32 | 0.99 | 16641 | 157 | 188 | 2.68 | 1.00 |
| Z1-2 | 6272 | 218 | 277 | 3.4 | 0.99 | 16641 | 119 | 142 | 2.36 | 1.00 |
| Z1-3 | 6272 | 230 | 284 | 3.69 | 0.99 | 16641 | 106 | 142 | 2.21 | 1.00 |
| Z3-1 | 6272 | 136 | 207 | 2.05 | 0.99 | 16641 | 100 | 154 | 2.2 | 1.00 |
| Z3-2 | 6272 | 193 | 232 | 3.67 | 0.99 | 16641 | 77 | 96 | 2.15 | 1.00 |
| M1-1 | 6272 | 234 | 350 | 3.49 | 0.99 | 16641 | 62 | 93 | 2.11 | 1.00 |
| M1-2 | 6272 | 222 | 308 | 3.62 | 0.99 | 16641 | 131 | 142 | 2.52 | 1.00 |
| M3-1 | 6272 | 193 | 248 | 3.14 | 0.99 | 16641 | 66 | 78 | 2.11 | 1.00 |
| M3-2 | 6272 | 117 | 191 | 2.51 | 0.99 | 16641 | 102 | 116 | 2.3 | 1.00 |
| P1-1 | 6272 | 161 | 189 | 3.42 | 0.99 | 16641 | 67 | 90 | 2.1 | 1.00 |
| P3-1 | 6272 | 205 | 253 | 3.51 | 0.99 | - | - | - | - | - |
| P6-1 | 6272 | 122 | 155 | 2.42 | 0.99 | 16641 | 120 | 135 | 2.37 | 1.00 |
| **Diseased** |  |  |  |  |  |  |  |  |  |  |
| W0 | 6272 | 211 | 236 | 3.61 | 0.99 | - | - | - | - | - |
| Z1-1 | 6272 | 88 | 107 | 2.2 | 1.00 | 16641 | 147 | 184 | 2.55 | 1.00 |
| Z1-2 | 6272 | 184 | 208 | 3.84 | 0.99 | 16641 | 115 | 134 | 2.72 | 1.00 |
| Z2-1 | 6272 | 189 | 229 | 3.47 | 0.99 | 16641 | 95 | 132 | 2.26 | 1.00 |
| Z2-2 | 6272 | 165 | 188 | 3.08 | 0.99 | 16641 | 66 | 95 | 2.12 | 1.00 |
| Z3-1 | 6272 | 209 | 256 | 3.9 | 0.99 | 16641 | 91 | 127 | 2.29 | 1.00 |
| M3-1 | 6272 | 131 | 166 | 2.48 | 0.99 | 16641 | 99 | 137 | 2.5 | 1.00 |
| P1-1 | 6272 | 148 | 171 | 3.15 | 1.00 | 16641 | 83 | 97 | 2.29 | 1.00 |


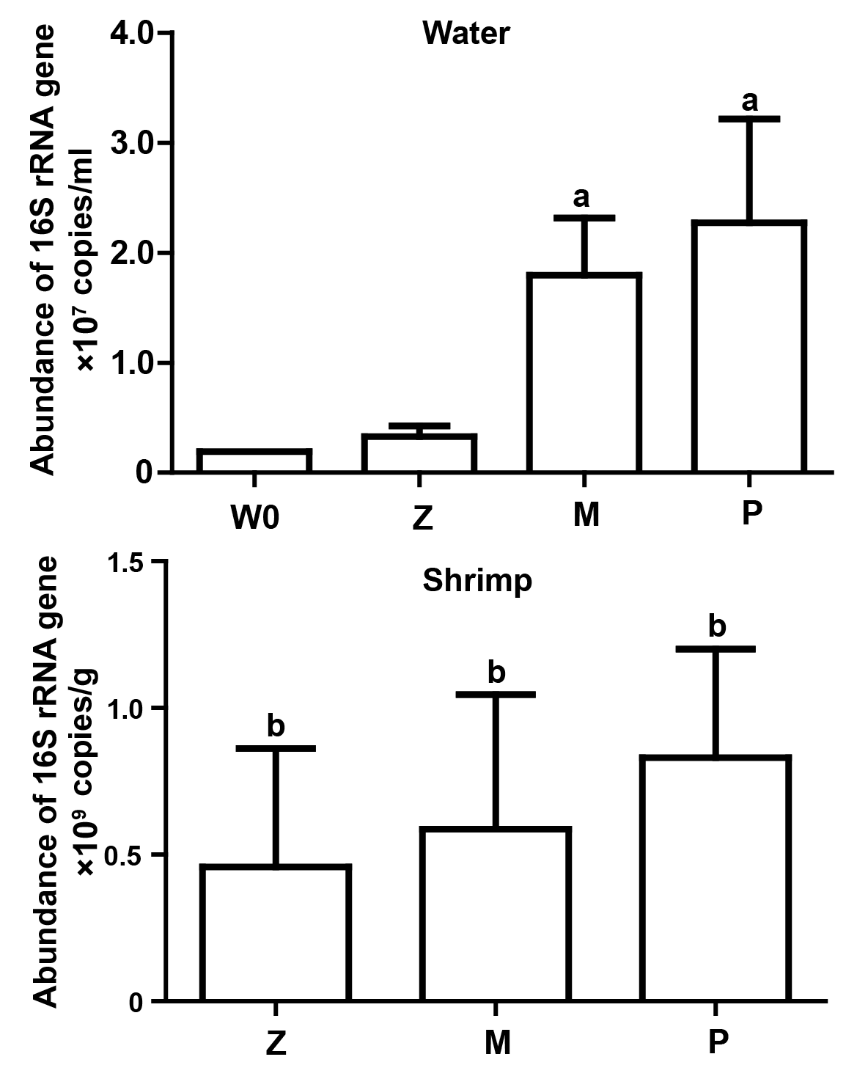


Fig. S1 Abundance of 16S rRNA gene in water and shrimp at different gowth stages. The same letters above the bar indicate there are no significant differences by *t* test. W0: water before larvae were released into pond. Z: zoea; M: mysis; P: postlarvae. Error bar: SEM.


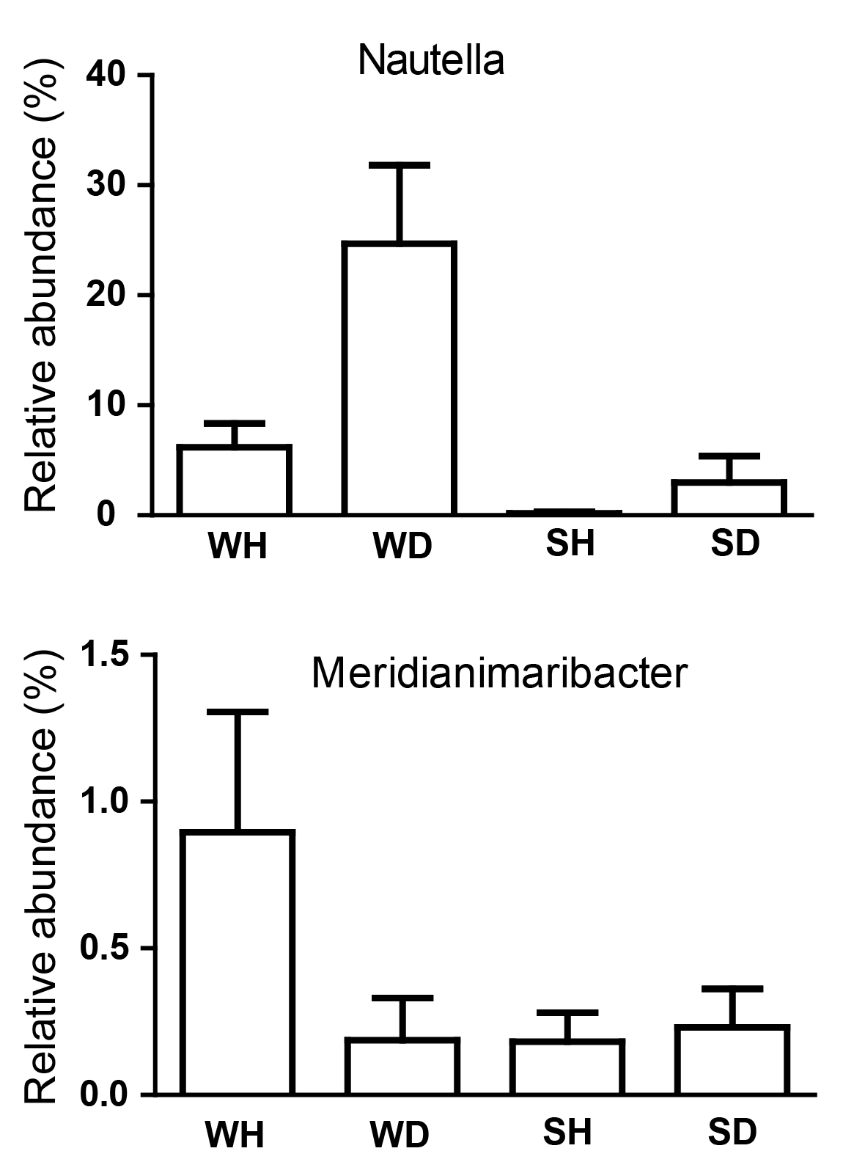


Fig. S2 The relative abundance of *Nautella* and *Meridianimaribacter* in WH, WD, SH and SD. WH: water samples from ponds with healthy shrimps, WD: water samples from ponds with diseased shrimps, SH: shrimp samples from ponds with healthy shrimps, SD: shrimp samples from ponds with diseased shrimps. Error bar: SEM.


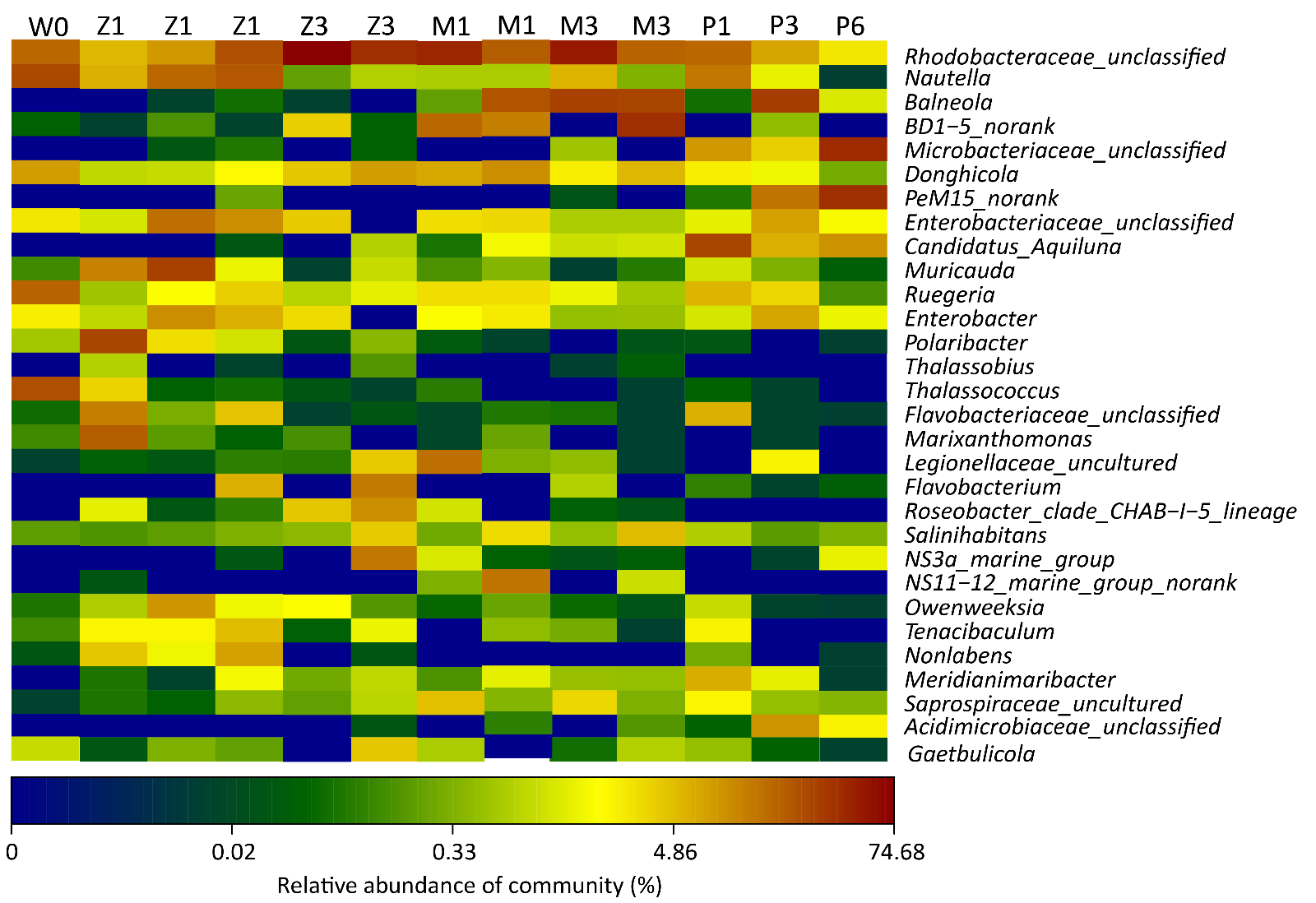


Fig. S3 The heatmap showing the top 30 abundant bacterial genera across healthy water. W0: water before larvae were released into pond. Z: zoea; M: mysis; P: postlarvae.
